# How Well Are Manufacturing Companies in Transylvania, Romania Adapting to the Low-Carbon Economy in Order to Become Sustainable?

**DOI:** 10.3390/ijerph19042118

**Published:** 2022-02-14

**Authors:** Mihai Dragomir, Diana Alina Blagu, Sorin Popescu, Mircea Fulea, Călin Neamțu

**Affiliations:** Department of Design Engineering and Robotics, Technical University of Cluj-Napoca, 400020 Cluj-Napoca, Romania; sorin.popescu@muri.utcluj.ro (S.P.); mircea.fulea@staff.utcluj.ro (M.F.); calin.neamtu@muri.utcluj.ro (C.N.)

**Keywords:** low-carbon economy, carbon footprint, sustainable manufacturing, new product development, process improvement

## Abstract

This paper addresses the degree of readiness of manufacturing companies in the well-defined area of Transylvania, Romania for tackling the challenges of the low-carbon economy (LCE) in view of the ambitious goals of the European Union. The presented survey aims to provide a better understanding about the management and reduction in the carbon footprint among production companies in Romania, as this sector is not usually included official strategies or studies. One hundred and three companies, selected based on voluntary sampling responses, were investigated using a 40-item questionnaire. The survey was applied to the manufacturing sector, including automotive, furniture production, and metal processing domains, which are locally representative and a good approximation of what small- and medium-sized firms look like across the EU, thus furnishing a good view of what takes place in other countries as well. The results obtained were analyzed using univariate descriptive statistics, multi-variate item analysis, and hypothesis testing to arrive at conclusions with a high degree of applicability. The purpose was to obtain an accurate overview about the actual situation and help companies find solutions in order to reduce the carbon footprint in the production field and achieve sustainable manufacturing. We arrive at the conclusion that manufacturing firms have a high degree of interest in decarbonization, but understand the efforts required to the same degree they understand the opportunities it brings. For example, 55% of respondents are interested in the benefits of LCE, while 90% of them observe at least one environmental standard, and ca. 70% implement at least common decarbonization measures, such as electricity savings or process optimization. While raising awareness and improving technological processes are accepted and embraced, other issues (i.e., involvement in RDI and CSR, change management, as well as financing investment efforts) should be addressed by proper policies.

## 1. Introduction

Reducing the carbon footprint by the manufacturing sector is a big step for many countries to align to the current polices promoted at the international level by the EU and the UN. The climate change problem is increasingly addressed as a climate crisis in the latest proposals by these international organisms, and actions to be taken in all aspects of the economy and society cannot be postponed any longer. As any consumer can see in the past couple of years, the banning of single-use plastic, the increased adoption of electromobility solutions, and improved energy use through LED lights and better insulation of buildings are already a reality, although they seemed improbable a few decades back.

The next frontier to be tackled is related to the carbon footprint, and one can observe it already when booking flights or choosing an energy provider; however, since CO_2_ has no color, no odor, and no taste, and our economy still depends on fossil fuels and naturally grown resources, the elimination of carbon should be as much a priority for governments and companies as it should be for individual persons. Many of the processes encountered during the lifecycle of producing and using a product can generate considerable amounts of carbon dioxide, in addition to other environmental impacts. For this reason, the manufacturing sector, especially related to items we use every day, such as cars or furniture, has a considerable impact upon the global carbon footprint and must be addressed in its own right, along with energy and transportation.

The first international agreement Romania took part in to decrease the carbon footprint was the Kyoto protocol [1], where signatory countries have committed to reduce emissions by 8% until 2012. Romania managed to reduce emissions and achieve the goal due to the fact that, after 1990, heavy industry disbanded and many polluting factories were closed. There was little to no effort made in the transition period of the 1990s to limit emissions of factories, and the good fortune of receiving credits in the carbon emissions trading schemes was largely wasted.

After this agreement, the most important is the Paris Agreement [2], which has the purpose to reduce the carbon footprint with 80–90%, until 2050, aiming for carbon neutrality or, at least, net-zero emissions. A very ambitious pact, signed by the almost all countries on Earth, to which Romania contributes and reports on a regular basis, this agreement could still be insufficient to address the challenges revealed by the latest IPCCC report [3].

Through the European Green Pact [4], the EU aims to become carbon-neutral by 2050, thus continuing its worldwide recognized environmental efforts and initiatives. This pact aims to step up efforts to combat climate change and make major improvements in line with the Paris agreement and beyond its ambitions, across all countries in the European Union and in all domains (energy, transportation, manufacturing, agriculture, etc.). Since most countries already submitted recovery and resilience plans to access European funding in the light of the COVID-19 crisis, the Green Pact should form the basis of most economic and industrial transformations to take place on the continent in the next 4 years. The funds available through this mechanism complement the structural funding already approved for the 2021–2027 financing period and the research and innovation funding available through the Horizon Europe program. With all these policies and resources in place, European countries, including Romania, where the current study was conducted, should be able to make important contributions towards a fully functional LCE.

Regarding the research results available on this topic, there are many studies that aim to analyze the state of certain countries about their carbon footprint, the internal mechanisms of processes or products needed to reduce the carbon emissions, as well as economic models that aim to increase the efficiency of the manufacturing factories, while at the same time becoming examples of LCE. Our literature survey presented below has not found significant studies in the manufacturing sector in Eastern European countries; however, the impact of environmentally blind industries in former communist blocks can still be felt in this area of new EU member states and can inform the local culture and actions to be undertaken. From this point of view, we consider our research approach novel and of potential interest for academics, company leaders, and policymakers in order to use the existing potential in meeting the above-mentioned goals, while avoiding potential pitfalls, redundancies, and waste of resources.

## 2. Literature Review

The scientific literature on the topic of decarbonization and LCE is growing at a fast pace in the past years, being divided into articles for the introduction and validation of specific technologies for reducing the carbon footprint, studies about country or sectoral responses to the ambitious objectives set out through policies, or survey studies of company practices and consumer preferences. This scientific niche is part of the larger body of literature oriented towards environmental studies, but it is quickly becoming a field of research on its own. For the purpose of preparing the survey of companies in Transylvania that is detailed in this paper, the authors have approached the literature of the most recent years (2019 to 2021) with search keywords in the ScienceDirect database, e.g., the terms “manufacturing” and “low carbon” (including some variations, such as “net zero carbon”, “carbon neutrality”, and “carbon footprint”), which best describe the objectives of the research endeavor. The same terms related to LCE (i.e., without “manufacturing”) were used for an additional documentation endeavor using Google Scholar in order to fill in the possible existing gaps in the first review pass and address related fields as well.

### 2.1. The Asian Perspective on LCE Transformations

The concept of LCE is first of all an overarching paradigm that should be implemented across a significant number of companies to produce the desired results. Usually, changing an entire economic sector requires heavy policy interventions and the modification of market conditions to be both achievable and sustainable. The study performed by [5] presents two economic models that aim to reduce the carbon footprint with both having certain merits and producing desired results. However, the researchers come to the realization that the usual way of improving low-carbon sustainable development performance is driven by technological progress through successive innovations, but the model of promoting increased production opportunities based on the existing damages to the environment that need to be overturned proves more effective in reducing carbon emissions. Manifesting these opportunities should come from a mix of strategic decisions and attractive profit opportunities, something we assumed Romanian companies are also looking for.

The analysis of the performance of Chinese manufacturing companies in the context of adopting low-carbon technological innovations [6] showcases other important insights in the studied area. The mentioned article reveals that large companies are self-motivated to employ innovation activities, while small- and medium-sized companies need an incentive from stakeholders to invest in new technologies. In order for these companies not to lag behind large companies, governments, especially in developing economies, should support them, either through subsidies or tax cuts, and/or financing adequate investment projects.

By rounding the mapping of the situation in China, one must notice that high-level LCE interventions also include the niche of labeling products with the carbon footprint they generate in order to impact consumer behavior at macro-economic level, for all industries, including manufacturing [7]. This empirical study shows that 86% of the people surveyed in China would accept an increase in the price of products manufactured under carbon label conditions, with the rest believing that the government is responsible for bearing such costs. Unfortunately, for Romania or other Eastern European states, such a study is not yet available.

In another developed Asian economy, in South Korea, a study by [8] investigated the disagreement and debate between the regulating authorities and the companies. By investigating 40 industrial sub-sectors and studying their performance over 6 years, the authors conclude that, although the government has adopted early policies, their effect is very small and should be changed based on a cultural shift at producer, consumer, and policymaker levels. We found this study relevant to our situation through its conclusions, as one of the working assumptions when developing and analyzing the current questionnaire was related to cultural factors pertaining to the environment in the Romanian economy.

In Taiwan, the research undertaken by [9] finds that the role of the government in the economy’s efforts to reduce the carbon footprint by means of carbon offsetting should be increased, with more institutions and more instruments to support the initiatives of the companies. The main conclusion that we appreciate for substantiating our own approach also is the need to enhance the public–private partnership in the field in order to achieve viable results.

### 2.2. The European Perspective on LCE Transformations

At the EU level of intervention in the national economies of its member countries, a study of the citizens’ views on carbon taxes in Europe performed by [10] identified a strong sense of personal responsibility on the part of people in trying to reduce the impact of climate change. However, according to the authors, citizens’ trust in political actors is an even stronger condition that opposes the sense of social responsibility, and this is in dispute in the post-modern era, especially since the COVID-19 pandemic started. Furthermore, they conclude, with regard to the acceptance of carbon taxes by the population, that it is quite difficult in the current context. As for Romania, where our survey was conducted, the situation would be even more complex, considering the low level of social trust between the authorities and the people, as seen in the poor results of the vaccination campaign [11]. An increase in the carbon tax to reduce emissions is not a measure that would be embraced well by the population. For example, in Romania, indirect carbon taxes are paid in electricity, gas, and other utility bills (e.g., high-efficiency co-generation contribution and green certificates for electric power at the national level imposed and calculated by the National Authority for Regulation in the field of Energy, local taxes for individual gas-powered central heating systems as opposed to building-level systems, service surcharge for unsorted waste applied by the waste service providers, etc.), but they are collected, and little change can be seen by the regular (home) customers. Greater transparency regarding the implementation of actions, as well as better dissemination of the concrete results achieved, would help in changing public opinion on these matters.

The study by the British consulting firm Cooper [12] shows that the public opinion on actions to reduce the carbon footprint to a net value of zero by 2050 is centered around the idea that not enough is being done. At the same time, the respondents in this study show a high availability of lifestyle change, although they consider that it is the responsibility of governments to implement such changes, without presenting any additional cost to the population. The three main sectors identified by respondents to be a focus for sustainable measures are transport, energy, and industry. This study highlights one of the motivations of the authors of the present paper, i.e., to investigate manufacturing as a rather neglected sector, although consumers realize its potential impact on climate change.

A study conducted in Germany [13] sheds light on a more subtle aspect, which can have important implications, especially in Europe, where 26 countries have different approaches to decarbonization. The authors discover that the commitment to this effort, including its costs, can vary from one economy to the other and has the potential to generate conflicting diplomatic opinions. In our case, with Romania being among the countries with a rather negative track record in adopting EU measures and making complex investments, we decided to include questions in the questionnaire about the general approach in the country to environment and the low-carbon economy.

An article by [14] investigates low-carbon technologies in Europe, i.e., a more technical dimension of diminishing carbon emissions. Three types of loop systems are analyzed. These types of systems have proven to be very effective in developing low-carbon processes and improve the efficiency of existing production technologies.

However, we consider that technology-based approaches are difficult to implement in Romania in small- and medium-sized companies for several reasons, including an insufficiently high level of awareness to actively seek the implementation of such systems, the need for further encouragement by policymakers, and the lack of pressure from the consumers upon companies to generate products and services in such systems. Consequently, even if the technology is developing globally and becoming more effective and efficient, its implementation actually depends on the issues investigated by the other studies, including consumer perception and fiscal policy acceptance.

### 2.3. Global Approaches to LCE Transformations

In the context of literature review, the global perspective can be based on professional publications, related to initiatives for decarbonization and their results, going back for a few more years than the recent scientific articles analyzed in the previous sections. These present projects and mechanisms are implemented by political actors in order to reduce the carbon footprint in more than one country. They bring interesting aspects to light, regarding the chances of success in Romanian companies in the near future.

The “Pathways to Deep Decarbonization project” [15] is an initiative that identifies ways in which countries can reduce their carbon footprint in an attempt to limit global warming. It started in the USA; however, since the beginning, the project has been scaled to many other countries and can be further expanded in the future. An analysis of the costs and technological changes, as well as their implications for achieving the goal of significantly reducing carbon emissions compared to pre-industrial levels, is also needed for Romania as soon as possible, as there is a lack of statistical information and a limited systemic approach to carrying out the activity of monitoring carbon emissions at the country level.

From a social perspective, the “Decarbonizing Development: Three Steps to a Zero-Carbon Future” initiative [16] emphasizes three fundamental principles by which the goal of reducing carbon emissions should reach the internationally assumed threshold: planning, carbon pricing, and social protection. Although plans to reduce carbon emissions exists in Romania, the application of measures similar to the other two recommend in this report still remains somehow unclear, at least until the implementation of the National Recovery and Resilience Plan. For the other partner of the social contract, the private sector, the “Product Carbon Footprint for Beginners” project [17], aims to provide a simple framework for producers to better understand the processes, challenges, and benefits of the market, as well as the impact that these activities can have on the environment. This direction too is investigated in the current survey, as the authors consider it the starting point of carbon awareness.

When approaching manufacturing as a stand-alone economic sector, we should still frame it considering its determinants, even when these adjacent domains are also studied separately, such as energy production to achieve a low-carbon footprint [18], educational approaches to create the necessary skills for LCE [19], SME competitiveness [20], youth involvement in the socio-economic transition to LCE [21], and the overarching cultural framework that determines the adoption of this paradigm [22].

Finally, by analyzing the body of knowledge presented above as a whole, the authors believe that the relation between industrial manufacturers, energy producers, and consumers, as investigated in [23], to which we would add logistic companies, could form the basis of a successful implementation of LCE in the future.

## 3. Materials and Methods

### 3.1. Localization and Time Frame

The quantitative research method employed was an opinion and awareness survey based on an online questionnaire that was sent by email through manufacturing companies from Transylvania and promoted through social networks (LinkedIn and Facebook) and implemented through voluntary participation. These characteristics were chosen to give the survey a higher chance of being answered by relevant stakeholders in the time period coinciding with the 2nd wave of the pandemic in Romania.

### 3.2. Survey Participants

The survey was answered by 103 companies from Transylvania, Romania, which were all active in the manufacturing sector in the following domains: automotive, metal processing, furniture, and a catch-all category of “other productions”. The method employed for sampling was based on a voluntary response, and the grouping into the above categories reflects the consultancy experience of the authors, which corresponds to the economic distribution reflected by the official strategies on a local level [24], though this is relevant at a European level too.

The collection period spanned 7 months, 2 of which were not active as holiday months (see the Results section for details). Data collection was stopped when the response rate became less than 1 response every day, calculated for 5 consecutive days, which can be considered to signal a depletion of discretionary interest because the time interval between responses was higher than the average lifespan of social media posts on the employed platforms, Facebook and LinkedIn [25]. At this specific time, the total number of answers was determined.

### 3.3. Research Method

To perform this survey study, the authors created a questionnaire with 40 items, using a mix of question types [26]. Single-choice questions had a direct target characteristic to investigate, while multiple-choice questions were used to define the frequency of occurrence regarding the preference for some input. In addition, the questionnaire contained evaluation questions using both nominal and ordinal scales. The nominal questions were used to classify the responded companies, such as the company size, the activity field, etc. In order to evaluate the responses, an ordinal scale was used to prioritize the investigated stimuli according to a certain criterion. The method used was the semantic differential, a scale with 5 levels between two opposite poles, thus measuring the intensity of an opinion/appreciation/preference regarding a certain stimulus. This technique allows for the best capture of the motivation of the company representative, which corresponds well to the current situation in the target country related to decarbonization, relying considerably on voluntary actions and investments. Before being sent out, the instrument underwent appropriate pretesting [27], from conceptual and technical points of view.

The questionnaire was divided into 4 different sections; the first one was for identifying the company’s field of activity; the second one was for assessing the environmental approach of the firm and general level questions about corporate environmental behavior in Romania; the third one was for describing the relationship with the specific legal and regulatory framework; and the fourth one was for the detailed evaluation of the carbon footprint reduction measures at the company, process, and product level.

### 3.4. Data Processing

The collected data were primarily interpreted through the means of descriptive statistics. Thus, the analysis performed by the authors was based on their experience in the manufacturing sector and the graphical representations that can be generated from the collected data. Additionally, well-known indicators, including the average and the sum, were appropriately introduced in the discussion. For a better understanding of the behavior of the studied companies with respect to environmental challenges and decarbonization, comparisons were made between subsets of the data collected, based either on the specific domain of activity (e.g., automotive, furniture production, etc.) or between large and small firms. A multi-variate item analysis was also included to determine the coherence of the main directions of company intervention.

The questionnaire implemented by authors can also allow for the determination of the truth status of a number of alternative hypotheses formulated on the basis of the observed behavior of targeted companies and industries. In this sense, if we formulate a generic null hypothesis stating that:

**H_0_.**  *Manufacturing companies in Transylvania interested in [topic] are as prepared as any company to get involved in the [action] environmental decarbonization effort.*

And contrast these with the data collected from more of the questions in the proposed survey, we can calculate the *p*-value for each of them, arriving at aspects (actions possible for a company) that are similar across the entire economic environment and aspects that are particular to the manufacturing sector.

## 4. Results

**Figure 1:**
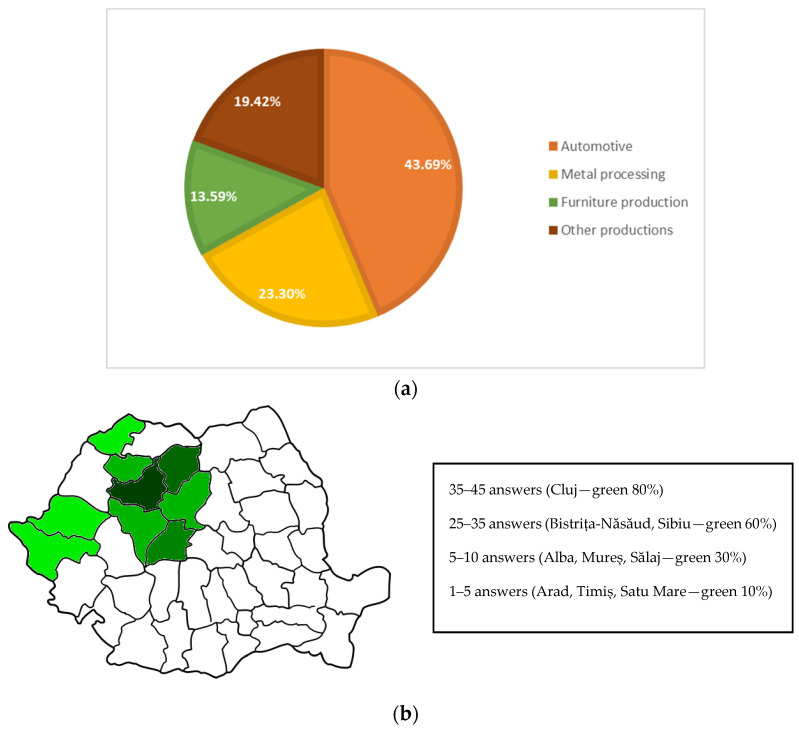
(**a**) Fields of activity of the companies surveyed. (**b**) Geographical distribution of the answers (hue corresponds to the number of answers).

The survey registered 124 answers, of which 103 are from the manufacturing industry (responses from other sectors have been eliminated). In this article, we aim to describe and interpret the answers from manufacturing companies due to the authors’ interest in the state of this field, while the others were used to provide the regional context. The manufacturing firms are split into the automotive sector, the metal processing sector, furniture production, and other types of production (such plastics, appliances, labels, etc.). As it can be seen in Figure 1a, there are 45 answers from automotive companies, 24 from metal processing, 14 from the furniture production sector, and the others account for 20 answers. The survey was applied between October 2020 and April 2021 in two phases. In the first part of the questionnaire distribution, referring to the first two months, we obtained 83 answers, among which 66 firms were from the manufacturing industry, while the other 17 answers were from other sectors, such as services, consulting, training, etc. Starting from February 2021, the questionnaire was resent to the focus companies. At the end of April 2021, the distribution of the questionnaire ended with 103 companies, whose field of activity was in the area of interest.

The geographical positioning of the companies that answered the questionnaire around Transylvania includes counties such as Cluj, Bistrița-Năsăud, Sălaj, Sibiu, and Alba, which contained some of the largest economic centers, with limited numbers from other counties in the region, such as Satu Mare, Arad, and Timiș (see Figure 1b). In sum, the analysis of this survey is clearly pinpointed in time and space, allowing for a better snapshot to be obtained.

**Figure 2:**
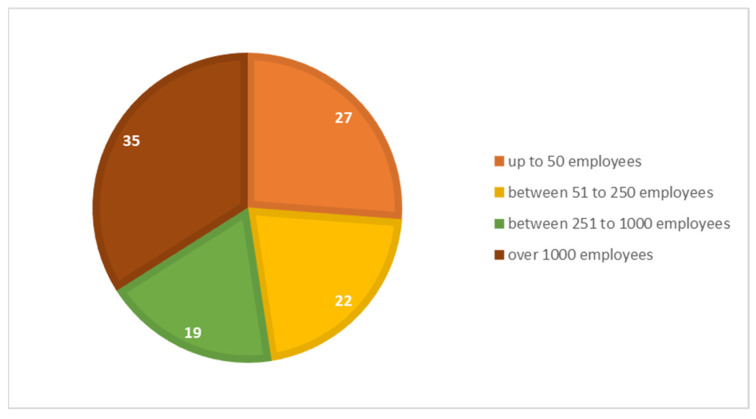
Size of the companies surveyed.

In terms of the company size, the surveyed organizations have a wide variation of the number of employees. As seen in Figure 2, the size of responded firms is diversified and balanced. This variety of the company proportions makes the interpretation of the results more relevant for the overall industry. The heterogeneity of the size of companies reveals a perceived average view on the actions needed and undertaken to reduce the carbon footprint. Furthermore, a comparative analysis of the answers will be performed depending on the size of the companies, divided into large and small companies. This comparative analysis will help us see the similarities and differences between them as there is a significant difference in the technology capabilities and in the accumulated know-how that they can employ during their efforts.

## 5. Discussion

All the answers will be interpreted as a whole, but an interpretation of the results according to the fields of activity of the companies is also made.

Regarding Romania’s overall level of training in solving environmental problems, the manufacturing companies are concerned that they will not be able to face the future challenges. The concern of the enterprises was measured on a scale with five levels, with one meaning not prepared and five meaning very well prepared. The average obtained was 1.99 which means that solving environmental problems in Romania is perceived as quite difficult. The comparison between small and large firms is insignificant in this case, as small companies obtained a score of 1.98 and large companies obtained a score of 2.02. Regarding a comparison between fields of activity of the companies, the scores obtained in descending order are as follows: other production fields—2.2, metal processing sector—1.93, automotive sector—1.93, and furniture production—1.71. Furniture manufacturers, who are directly processing natural resources for their products, have the lowest opinion on this issue.

**Figure 3:**
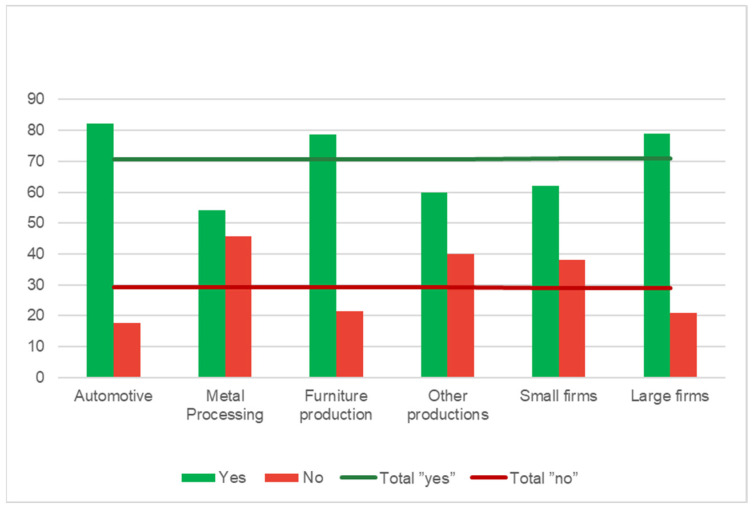
Companies that select partners based on environmental issues (percentage of answers).

Regarding the selection process of stakeholders to whom they collaborate with, 71% of respondents take into account the attitude towards the environment of the interested parties. In Figure 3, the average of the positive answers is marked with a green line and the average of the “no” answers is marked with a red line. Additionally, in Figure 3**,** the answers are presented for each sector of the responding firms. The green bars represent the answers of the companies that take into account the attitude of stakeholders about the environment and the red bars represent the companies that do not take this into account.

All the answers are represented in percentages, and it can be seen that the automotive and furniture production firms are more careful about the companies they work with, as both green bars exceed the green line while both red bars are under the red line.

In addition, the same graph represents the answers from small and large companies. As it can be seen in the figure, the large firms are more open to choosing their collaborators based on their concerns with the environment (more “yes” answers than small entities).

**Figure 4:**
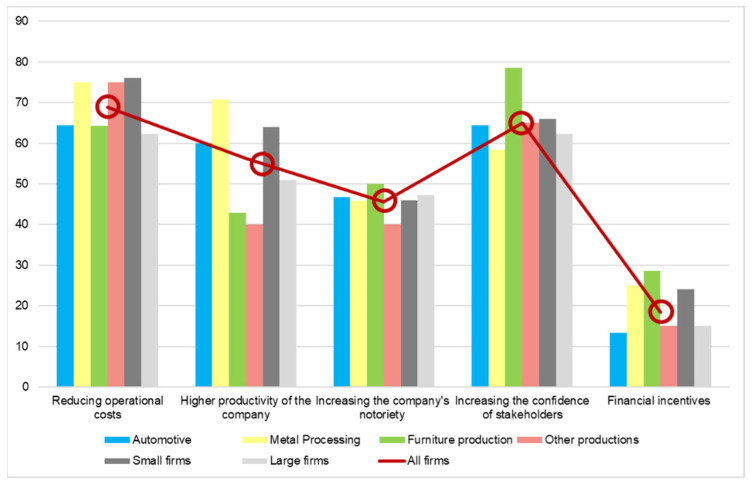
Benefits targeted by firms implementing environmental measures (percentage of answers).

In the next area of the questionnaire, they were asked about the benefits they aim for when implementing the recommended actions for protecting the environment. As it can be seen in Figure 4, the red line marks the trend of all the companies answers and the bars represent the answers divided by the field of each company as well as the size of the companies, split into small and large firms. All the measures on the graph are expressed in percentages. So, as it can be seen, the three main benefits targeted by the respondents are reducing operational costs, increasing the confidence of stakeholders in the company’s products, and higher productivity of the company. All of these benefits are targeted by more than 55% of all the manufacturing firms which answered the questionnaire. Besides, financial incentives are of less benefit, as targeted by all the companies. The targeted benefits by automotive firms, above the average, are higher productivity of the company and increasing the company notoriety, while the others were slightly below the average. In Figure 4, the automotive sector is represented by blue bars. For metal processing, the yellow bars, and the benefits targeted by them above the average, represent the reducing operational costs, higher productivity of the company, and financial incentives. As seen in the figure, the difference is more significant than in the automotive sector, where the differences are not easy to notice, mostly because they are part of large international conglomerates with well-defined environmental policies. For the companies from furniture production sector, increasing the confidence of the stakeholders, financial incentives, and increasing the company’s notoriety are the benefits targeted above the average. Additionally, it can be seen in the figure that, for this sector, the confidence of stakeholders is very important. The furniture production firms are marked with green bars. The other production sector is close to the average, which is to be expected in a mixed category, with only the productivity of the company differing, which is highly below the target.

Between small and large firms, the differences are seen for two of the benefits, i.e., reducing operational costs and higher productivity of the company, with large firms showing more interest and dedication in comparison with small firms. Furthermore, both of them are quite close to the average since they agglutinate various activity domains.

**Figure 5:**
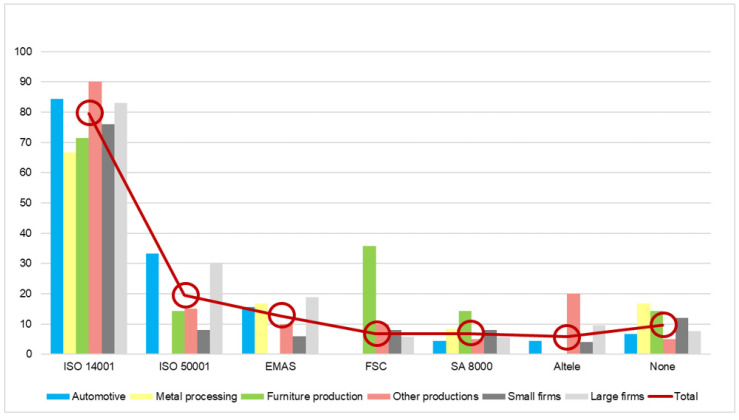
Environmental standards implemented by manufacturing firms (percentage of answers).

Moreover, Figure 5 presents the standards regarding the environment which manufacturing companies are working with. The environmental management system ISO 14001 is the most implemented standard by the manufacturing companies questioned. The important thing here is that only 10% of the questioned firms have no standard implemented. In addition, 90% of them have at least one standard implemented and 80% have ISO 14001 implemented, which is considered the first important step for companies to adopt the right measures for environment by many. This fact represents a good start for manufacturing companies to invest more in their processes and products and make them more friendly to the environment. However, the opinion of the respondents regarding the impact of implementing these standards is not as positive. On the following question, this attitude is measured on a scale with five variables, where one means not helpful and five very helpful. The average obtained is 3.50, which means that more than a half consider that these standards are helpful for the environment. From this, two conclusions can be drawn. Either the respondents consider that one must do more in order to achieve the current environmental targets of the European Union, or the implementation of these standards is not well done, and the results obtained are not the results that they expected.

**Figure 6:**
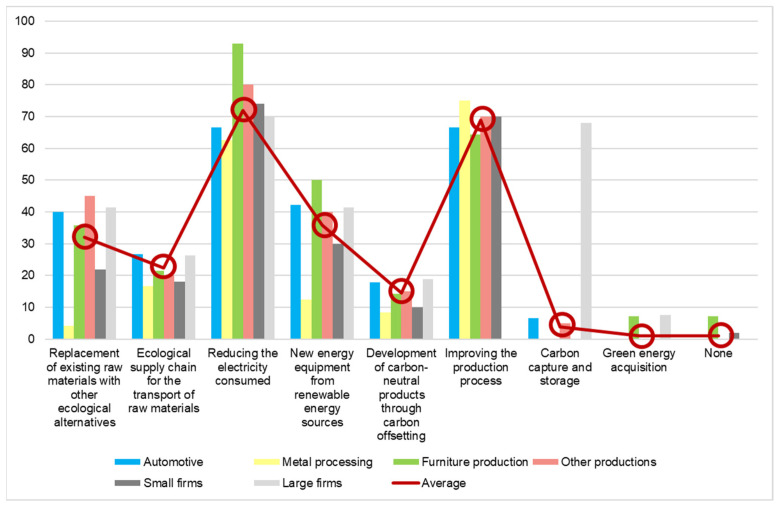
Measures implemented to reduce the carbon footprint (percentage of answers).

The most implemented measures in order to reduce carbon footprint include reducing the electricity consumed by using ecological lightning fixtures, investing in state-of-the-art technological equipment, and improving the production process by ensuring increased efficiency and eliminating downtimes. The percentage of the companies which implement these measures is ca. 70%. As it can be seen in Figure 6, the red line shows the average expressed in percentages of the measures implemented by the studied companies. Furthermore, there are only nine companies which do not take any measure to reduce the negative impact on the environment, which is less than 1% of the total. In second and third place, the survey revealed process improvement, which is analyzed in detail further in this section, and raw material replacement, respectively. This is line with the current findings at the European level that have been presented in the study [28] of material supply chains. Since the companies come from different parts of the value creation process, we consider that the market is already aware, perhaps in a subconscious form, of the potential of carbon emission reduction identified by the mentioned study. Further stimulation and support from the public sector could contribute to transforming this potential into actual savings, and these measures should take into account the conclusions of [29] about pricing practices being more relevant than technology implementation.

Even if the other measures are not so frequently implemented by the manufacturing companies, there is a solid foundation of environmental management practice to build upon. It is also possible that the purpose of the firms is to increase the production capacity and to better manage their resources, in order to stay competitive; however, if these economic concerns are properly interwoven with the environmental policies by the authorities, both sides of the issue can be successfully accomplished. Companies can survive on the market and the carbon footprint can diminish. Another researched topic within the questionnaire was to find out if the companies are involved in any type of carbon footprint reduction projects and the answer of 33% of the companies was no, which indicates there is significant room for development and improvement in the future.

The product development and design stages are critical for achieving low-carbon performances as mentioned by [30] in their study about the sand-casting process. In order to investigate this feature of LCE manufacturers and to find out which is the most frequently used technique in developing new products, a question with multiple choices was used with the following choices: stage gate model, design for six sigma, and innovation funnel. The stage gate model is a technique that has the purpose to transform the development process into sequential steps, and validate each of them, before proceeding further, from the idea of the new product to its launch on the market [31]. Design for six sigma is a tool for implementing a highly disciplined customer-centric methodical way for the design and development of products or services [32]. An innovation funnel is a tool used to transform an idea from the beginning of the creation process into reality by eliminating nonviable contributions along the way. The graphic form of the tool is a converging funnel which starts with the collection of all the inputs in order to investigate the feasible ideas, selecting the best of them and developing them to the point to deliver the final product [33]. In our study, a determination of CO_2_ reduction was not targeted, as was the case of [30], but the general list of development algorithms and tools can support companies in making their own assessments in terms of the effectiveness of employing these approaches to their own specific sub-sector of manufacturing.

**Figure 7:**
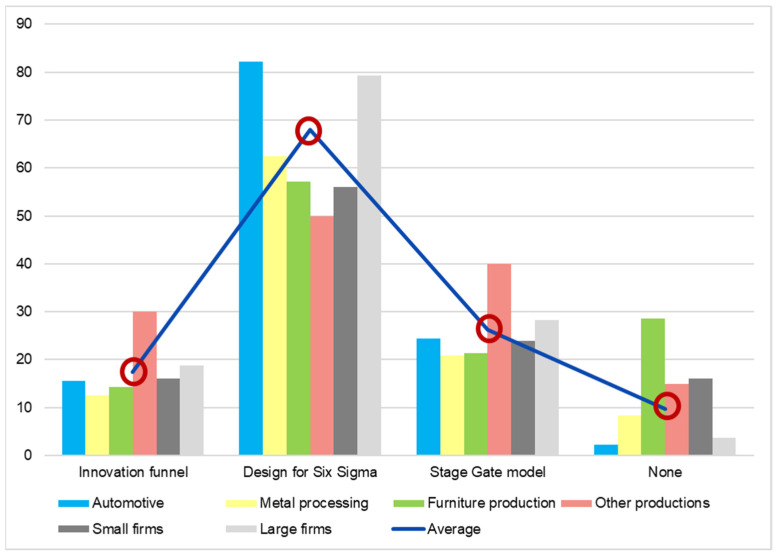
New product development techniques applied (percentage of answers).

As seen in Figure 7, the most frequently used technique is design for six sigma with the two variants of its processes: the DMADV (Define, Measure, Analyze, Design, Validate) methodology and the IDOV (Identify, Design, Optimize, Verify) methodology. This technique is implemented, in one form or another, by almost 70% of the respondents of the questionnaire. The other two techniques are implemented by a percentage between 20 and 30% of the firms. The worrying results are related to the companies from the manufacturing field which have not implemented any of these techniques. There is a proportion of 10% of the manufacturing companies questioned which falls into this category. This alludes to the problematic future of the companies because they will find it difficult to adopt carbon emission reduction approaches at the product level.

As also seen in Figure 7, the trend of not developing the new products in a structured manner is specific to small companies. The dark grey bars represent the small firms and the light grey bars represent the large firms. The trend for applying techniques to improve is higher for each choice for large firms, except the nonchoice, where the bar of the small companies is higher. Moreover, the graph shows that automotive companies are the most involved companies in using validated algorithms to develop new products.

In order to find out which is the most frequently used method for improving the processes, the questionnaire applied a question with multiple choices, such as lean manufacturing, Kaizen, and Six Sigma. These are well-known approaches, applied on a large scale in production companies all over the world (see [34] for details), and the Romanian companies answering the questionnaire were also familiar with them.

**Figure 8:**
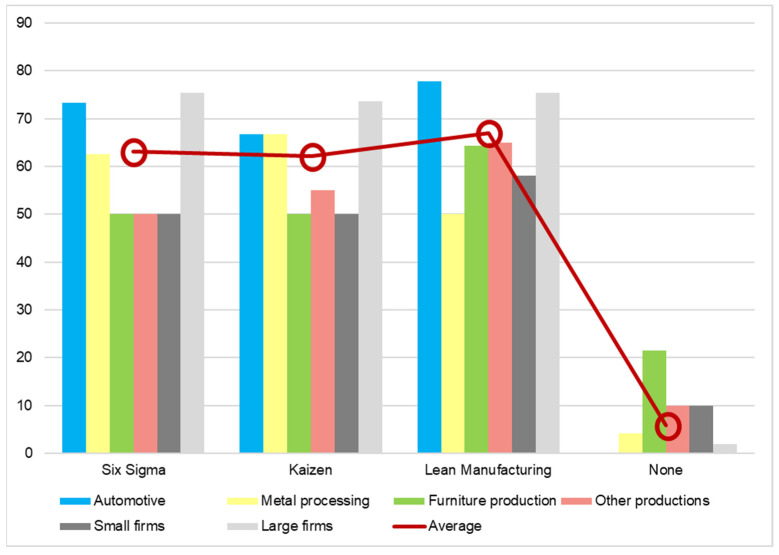
Process improvement methods applied (percentage of answers).

As seen in Figure 8, the most frequently method used is lean manufacturing which aims to reduce losses and waste. Additionally, when processes are concerned, the manufacturing companies applied more methods to improve processes than to develop new products. There are only seven companies which implement no method in this regard.

The conclusion that can be formulated regarding the techniques for developing new products and the process improvement methods is that manufacturing companies want to invest more to keep processes under control and to minimize losses, as opposed to developing new and more carbon-efficient products. However, minimizing waste, keeping variability low, and continual improvement are also helpful for the environment and reducing carbon emissions, but it might be insufficient since the products themselves carry the potential to pollute and contribute to climate change. If Romania is to achieve the targets of European Union, it is important to make changes also in the kind of products that people buy and the consumption behavior [35], which is a long-term mission of both the public and the private sector.

**Figure 9:**
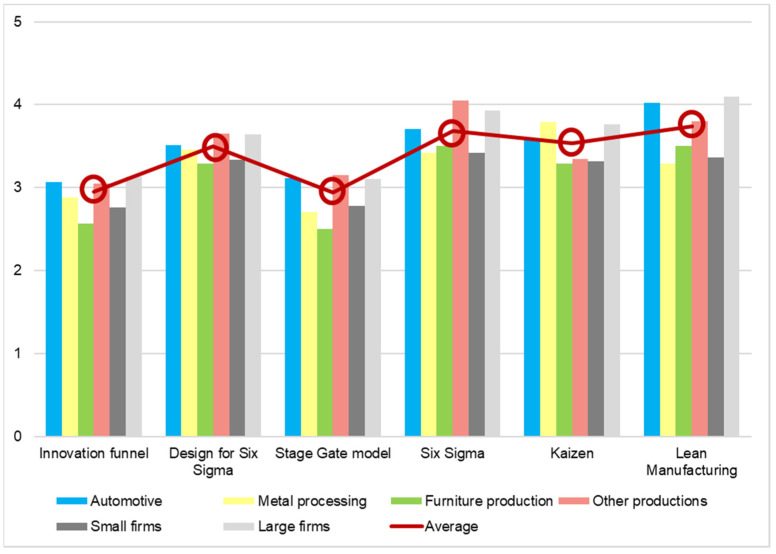
Helpfulness of techniques and methods in reducing the carbon footprint (average score).

Another topic researched in this questionnaire is evaluating the degree of helpfulness of the techniques and methods discussed above in diminishing the carbon footprint of the industry. These results can suffer some errors due to the fact that all the manufactured companies answered this question, but not all of them know or used one or more of these techniques or methods. However, the results displayed in Figure 9 are encouraging in the long term.

The techniques and methods were evaluated on a scale with five levels, where one means not helpful and five means very helpful. From the results obtained, lean manufacturing appears to be the best technique used in improving processes, while design for six sigma is the best method for developing new products. In addition, the grade obtained by them is 3.74 and 3.5, respectively, which is higher than the average, but still far from the top of the helpfulness scale.

**Figure 10:**
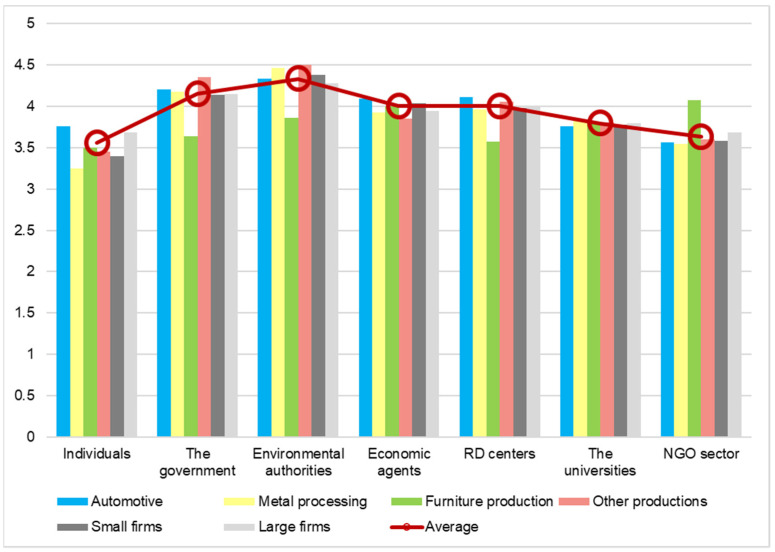
The importance of adopting measures by various stakeholders (average score).

The last question interpreted through this analysis is regarding the importance of the involvement of stakeholders in adopting measures to reduce the carbon footprint. The results show us that the manufacturing companies consider that the most important actor for implementing actions to reduce the carbon footprint are environmental authorities. They obtained a score of 4.34 on a scale with five variables, where one means low importance and one means high importance. The respondents consider that the next important actor for adopting measures in order to reduce the carbon footprint is the government. The score obtained by the government is 4.16, a little bit less than the environmental authorities. The third most important actor in this evaluation is economic agents. They obtained a score of 4, which means that the manufacturing companies consider that they play an important role in this domain. The smallest score obtained is by individuals. They obtained a score of only 3.55. All the results obtained by each stakeholder can be seen in Figure 10.

The fact that manufacturing companies consider that they themselves play an important role in reducing carbon footprint is a good sign for achieving the targets set by the European Union. In order to do that, if the attitude is passed on to each employee and they are aware of the importance of taking actions for reducing the carbon footprint, it would be easier to achieve changes in the personal lifestyles as well. The level of ambition in reaching the necessary goals will definitely require all economic sectors to contribute and the regular people to change their behavior as consumers. Decarbonization has the potential to change the way our society and economy works in the next 20 to 30 years, and manufacturing firms must play significant role.

**Figure 11:**
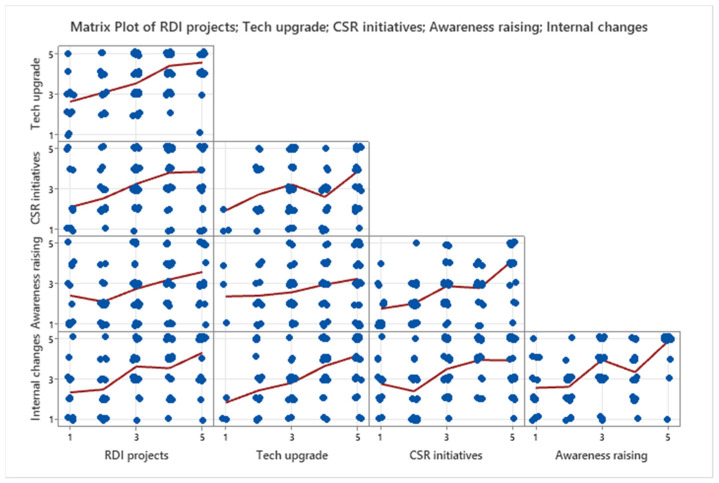
Correlations among the possible LCE conducive actions.

A multi-variate analysis was performed using item analysis implemented in the Minitab software upon five possible actions to facilitate the LCE transformation, This showed the following results (Figure 11).

With a Cronbach’s alpha coefficient of 0.7628, reflecting the internal consistency of the data, we can affirm that the possible measures support and complement each other. This is, however, not true for the four determinant topics selected from the literature (Cronbach’s alpha coefficient of 0.0507), which means that the social and economic expectations towards the companies are clear for them, but what they must actually do and how they can be supported is still a “fuzzy picture” for the private sector. It is possible that this confusion is even greater in the public sector in order to help the companies. Still, considering that there are possible punctual connections, we intend to perform another comparative investigation through statistical testing.

Null hypotheses testing has been performed using the collected data to study the degree of readiness of manufacturing companies to adopt LCE practices and to infer possible causes for the recorded results based on the formulated questions. The instrument used was the t-test [36], implemented in Microsoft Excel in a two-tailed and paired configuration. This was suitable for the existing data, relating interest of the companies in a LCE topic and the actions that they can undertake to bring about the expected decarbonization impact. The results obtained are presented in the table below (Table 1).

As such, we must surmise that the companies that declare their interest in low-carbon technologies and investment sources for them are already ahead of the transformation curve for LCE and will be in a better position than most other companies to implement technology upgrades in the near future, based on superior information and understanding (alternative hypothesis was validated as opposed to the “everybody is the same” situation). Another inference is that companies that understand the mechanism of CO_2_ production, related to their process, products, and raw materials (that desire to measure the carbon content and the calculate their own carbon footprint), already have a higher degree of awareness than their peers and are ready to spread this knowledge to their employees and to their partners, and possibly even share it with the society at large, keeping in mind that carbon dioxide is a global problem (alternative hypothesis also validated, in two cases).

## 6. Conclusions

The present survey shows that there are still many measures that factories from Romania have to implement in order to decrease their carbon footprint and participate in the new net-zero carbon economy of the European Union. In our opinion, firstly, the manufacturing industries from Romania should be more aware about the importance of this subject. There are many instruments which can not only help a company to be more efficient in this field, but can also help the company to implement these measures in general. Among these tools, it is important to mention the multiple standards which exist for reducing carbon emissions whose requirements, if fulfilled, provide a good practice guide for any company. Secondly, the adoption of low-carbon or no-carbon technologies and materials, as part of their approach to satisfying the customers, can yield improved ecological results during manufacturing and along the lifecycle of the product. As the survey showed, the companies are expected to invest in new technologies and develop the awareness of their personnel; however, regulations and policies are still needed to support these transformations. Manufacturing is one of the oldest and probably one of the most conservative economic sectors, so any change, even at such a prudent pace, is significant.

The overall view of the manufacturing industry landscape in Transylvania with respect to adopting low-carbon-emission practices is one of cautious optimism. On the one hand, the firms have evolved considerably in the past 20 years and became competitive on a European scale, while on the other hand their relationship with the environment is not completely defined. This is valid when discussing basic environmental protection measures, so when a new level of complexity is added by mandating climate change mitigation measures and low-carbon-economy approaches, the companies are confronted with a significant experience and support gap. It would be recommendable to improve the legislative framework, to direct funding in this area, and to leverage the existing know-how of universities in order to increase both general awareness and direct practical skills. By incorporating environmental topics in the engineering and economic sciences curricula in academia, and also by providing free information sources about CO_2_ reduction practices based on in-house company training for operational personnel, some important steps can be made by manufacturing companies in this direction in a reduced timeframe.

All these aspects can significantly benefit from the proper policy approaches that can be adopted at a national level in Romania and other EU countries. Such support should include grants and risk financing, but also fiscal measures such as tax incentives which would allow companies to invest in their environmental approach, as revealed in the current study, without compromising their competitiveness. At the current time, in Romania, there are programs aimed at decarbonizing the energy sector, residential heating, and transportation, but manufacturing is not specifically targeted, which represents a missed opportunity.

The study presented is limited in scope, as mentioned previously, and is also a voluntary survey which might suffer from confirmation bias, since companies answering are the ones already involved in environmental and climate actions. However, we consider that the content of the measures proposed for improving the sustainability results in manufacturing would be the same, even if this involves using a larger and more diverse sample of companies.

## Figures and Tables

**Table 1:** Null hypotheses testing among LCE topics and actions (accepted hypothesis indicated).

	Actions	RDI Projects	Tech Upgrade	CSR Initiatives	Awareness Raising	Internal Changes
Topics						
**Tech investment**		Null	**Alternative** ***p*-value = 0.566**	Null	Null	Null
**Regulation ambition**		Null	Null	Null	Null	Null
**Carbon content**		Null	Null	Null	**Alternative** ***p*-value = 0.091**	Null
**Voluntary footprinting**		Null	Null	Null	**Alternative** ***p*-value = 0.162**	Null
